# Lack of evidence for BCG vaccine protection from severe COVID-19

**DOI:** 10.1073/pnas.2016733117

**Published:** 2020-09-29

**Authors:** Cecilia S. Lindestam Arlehamn, Alessandro Sette, Bjoern Peters

**Affiliations:** ^a^Center for Infectious Disease and Vaccine Research, La Jolla Institute for Immunology, La Jolla, CA 92037;; ^b^Department of Medicine, University of California San Diego, La Jolla, CA 92093

It is important to understand the divergent incidence and impact of COVID-19 on different countries. A recent study in PNAS (1) hypothesized that bacillus Calmette–Guérin vaccination may protect from COVID-19 based on a negative correlation of bacillus Calmette–Guérin vaccination rates and COVID-19 mortality between countries, even after careful correction for cofactors such as population age, density, developmental state of the country, and bacillus Calmette–Guérin vaccination policies. It was concluded that bacillus Calmette–Guérin vaccination could have a protective effect from severe COVID-19 disease that might be mediated by trained innate immunity.

**Fig. 1.**
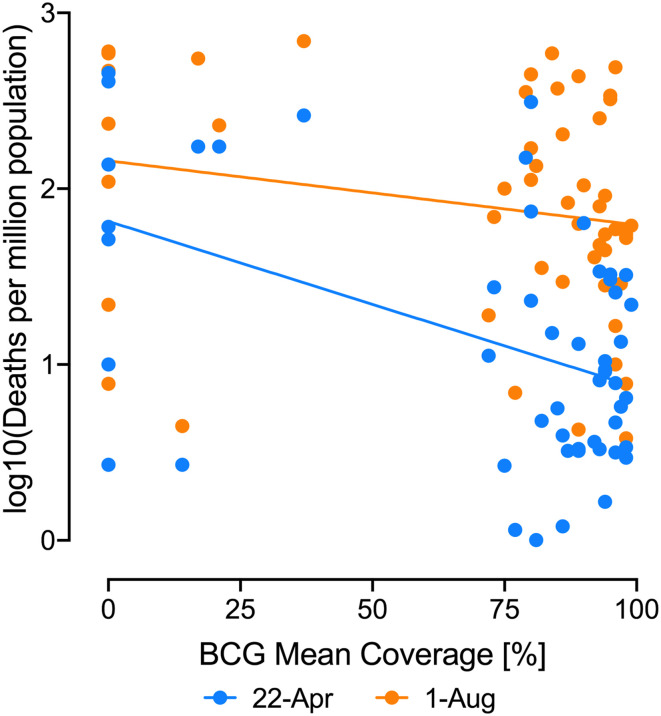
Association between percentage of vaccination coverage and maximum COVID-19 deaths per million inhabitants registered by country. Log_10_ deaths per million inhabitants vs. percentage of bacillus Calmette–Guérin vaccination coverage. Data from April 22, 2020, blue dots and line (Pearson *r* = 0.46, *P* = 0.0006), and data from August 1, 2020, orange dots and line (Pearson *r* = 0.2, *P* = 0.16).

The mortality data analyzed in ref. 1 covers the period until April 22. Since then, the pandemic has substantially shifted toward South America. We performed an updated analysis using dataset_S01 from ref. 1 to obtain homogeneous countries with Human Development Index of >0.7, >60% urban population, <300 inhabitants/km^2^, and >1 COVID-19 death/million on April 22 as in ref. 1. We did not exclude populations with <15% of age over 65, as that excludes all of South America (apart from Uruguay and Cuba). For the selected 51 countries, we found a significant negative correlation (*P* = 0.0006) between bacillus Calmette–Guérin mean coverage (1) and deaths reported on April 22 (Fig. 1; Pearson *r* = 0.46). However, when using updated mortality data from the Johns Hopkins database (2) from August 1, 2020, there was no longer a significant negative correlation (*P* = 0.16, Pearson *r* = 0.2). Importantly, the countries with the largest increases in deaths on August 1 are Bolivia, Panama, Columbia, Peru, Brazil, Mexico, and South Africa, which all have high bacillus Calmette–Guérin coverage, suggesting that current trends in the pandemic will further reduce the negative correlation between bacillus Calmette–Guérin and COVID-19 mortality.

**Fig. 2.**
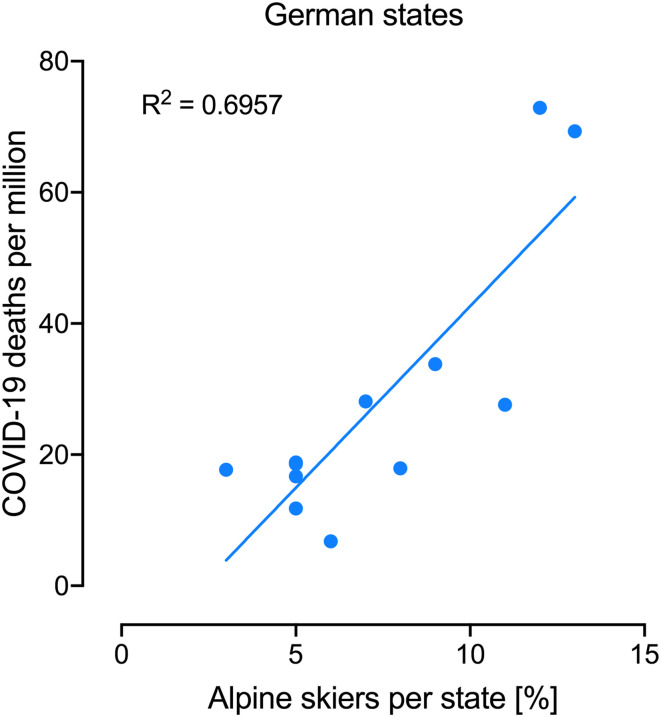
COVID-19 mortality rates correlates with the frequency of alpine skiers in different German states. Frequency of Alpine skiers was taken from ref. 10, and COVID-19 mortality in different German states was taken from ref. 1, excluding city states and the Saarland.

Another analysis in ref. 1 compares mortality in Eastern vs. Western states in Germany, suggesting that lower mortality in Eastern states could be linked with >60-y-old adults having received bacillus Calmette–Guérin in childhood, which was not the case in the West. This implies that childhood bacillus Calmette–Guérin vaccination has a 60+-y effect on trained immunity, which would be surprising. Heterologous effects mediated by bacillus Calmette–Guérin (3–6) through trained immunity (7) wane after a year following vaccination (8). The authors acknowledge other differences between East and West Germany besides bacillus Calmette–Guérin vaccination, but state that “it is hard to envision how these factors could decrease their risk of COVID-19 fatality in Eastern German states.” We suggest such an explanation, namely that the main spread of infections at the early stages of the pandemic in Germany was tourists returning from skiing vacations (9), and that the percentage of alpine skiers in different German states (10) correlates well with the reported mortality in ref. 1 (Fig. 2).

In conclusion, we believe that current mortality rates of the COVID-19 pandemic do not support a clear negative correlation with bacillus Calmette–Guérin coverage and that there are alternative explanations for the differences observed between Western and Eastern Germany states. Ongoing randomized controlled trials will provide answers to whether bacillus Calmette–Guérin reduces the incidence and severity of COVID-19 through its heterologous effects.
